# Construction of a vascularized fascia-prosthesis compound model with axial pedicle for ear reconstruction surgery

**DOI:** 10.3389/fbioe.2023.1126269

**Published:** 2023-05-24

**Authors:** Guanmin Li, Chen Lei, Xiuying Shan, Xuejun Ni, Guojie Chen, Meishui Wang, Ruonan Ke, Biao Wang

**Affiliations:** ^1^ Department of Plastic Surgery, The First Affiliated Hospital, Fujian Medical University, Fuzhou, China; ^2^ Department of Plastic and Wound Repair Surgery, National Regional Medical Center, Binhai Campus of the First Affiliated Hospital, Fujian Medical University, Fuzhou, China; ^3^ Senior Department of Burns and Plastic Surgery, The Fourth Medical Center of Chinese PLA General Hospital, Beijing, China

**Keywords:** ear reconstruction, vascularized tissue engineering chamber, pedicled fascial flap, ear prosthesis, abdominal superficial vessels

## Abstract

**Background:** To design a vascular pedicled fascia-prosthesis compound model that can be used for ear reconstruction surgery.

**Methods:** A vascularized tissue engineering chamber model was constructed in New Zealand rabbits, and fresh tissues were obtained after 4 weeks. The histomorphology and vascularization of the newly born tissue compound were analyzed and evaluated by tissue staining and Micro-CT scanning.

**Results:** The neoplastic fibrous tissue formed in the vascularized tissue engineering chamber with the introduction of abdominal superficial vessels, similar to normal fascia, was superior to the control group in terms of vascularization, vascular density, total vascular volume, and total vascular volume/total tissue volume.

**Conclusion:** *In vivo*, introducing abdominal superficial vessels in the tissue engineering chamber prepped for ear prosthesis may form a well-vascularized pedicled fascia-prosthesis compound that can be used for ear reconstruction.

## Introduction

As an important superficial aesthetic apparatus of the face, the external ear has a complex anatomy and functions to collect sound, protect the external auditory canal and eardrum, and enable the wearing of tools. Its superficial and protruding structure makes it highly susceptible to damage. In addition to causing much inconvenience, external ear defects can also have a negative psychological impact on the patient. The complex structure of the external ear makes ear reconstruction a challenging task for plastic surgeons (Brent, 1999; Steffen et al., 2010).

Ear reconstruction can be divided into two parts: the construction of the ear framework and the covering with soft tissues, while revascularization is an essential aspect of the reconstruction. Thus, obtaining vascularized soft tissue coverage is an important step in ear reconstruction. Patients with external ear defects due to trauma or burns or patients who undergo second ear reconstruction, these patients have a lack of soft tissue around the ear, which causes difficulties in surgery (Bos et al., 2016). The current clinical treatment options for these patients are diverse and include using adjacent soft tissue expander implantation, distant site full-thickness skin transplantation, and tubed flap shifting. However, these options still suffer from poor postoperative blood supply to the reconstructed ear, susceptibility to necrosis, swelling, and bad morphology (Washio, 1969; Achauer et al., 1991; Mutaf et al., 2006). Therefore, we considered the construction of a vascular pedicled fascia-prosthesis compound model that could be used for ear reconstruction surgery to provide a soft tissue coverage method for patients with peri-auricular soft tissue deficiency.

The “vascularized tissue engineering chamber” provides vascular support, nutrients, and room for tissue growth by inserting blood vessels into a tissue engineering chamber made of biologically inert materials with a certain degree of hardness (Hofer et al., 2003). This solves the problem that traditional tissue engineering products are difficult to develop and produce stable tissue constructs because nutrition can only be infiltrated by surrounding tissues (200–300 μm) (Mian et al., 2000; Dolderer et al., 2007; Eto et al., 2012; Kato et al., 2014; Dew et al., 2015; Mashiko and Yoshimura, 2015; Mayoral et al., 2022). We consider the construction of an *in vivo* vascularized tissue engineering chamber of fascia-ear prosthesis model for ear reconstructive surgery by introducing vascular bundles and pre-positioning a 3D-printed ear framework.

## Materials and methods

### Animals

4–6 weeks male New Zealand rabbits, weighing from 2.0 to 2.5 kg. The Animal Ethics Committee of Fujian Medical University approved animal experiments.

### Tissue engineering chamber

The tissue engineering chamber is made of medical silicone with a certain degree of hardness. The chamber is hemispherical in appearance, with an internal chamber diameter of 4.0 cm and a uniform distribution of holes of about 1.0 mm in diameter on the surface, and an opening of 1.0 cm × 0.5 cm on the bottom wall of the hemisphere for blood vessels to enter.

### Ear prosthesis

The ear prosthesis was sculpted according to the Nagata method, with a maximum diameter of 3.5 cm, and scanned and 3D printed to obtain the ear prosthesis. The above mentioned tissue engineering chamber and ear prosthesis are obtained by 3D printing with biocompatible resin materials (MED610, Stratasys Ltd.) (Mottini et al., 2016; Currens et al., 2022). The implants were sterilized using low-temperature plasma sterilization.

### Grouping and surgical protocols

Preparation: The rabbits were anesthetized with 3.0% sodium pentobarbital (1.0 ml/kg) intravenously through the ear margin, fixed in the supine position, and the lower abdomen, bilateral groins, and medial knee fur were removed.

Procedure: After skin disinfection, the skin (approximately 5 cm) was incised parallel along the groin, the deep fascia was separated, the inguinal fat pad was exposed, the fat pad was separated from the surrounding structures, and the branching small vessels were ligated using a 4–0 silk wire. The appropriate size cavity (about 5 cm × 5 cm × 5 cm) was bluntly separated between the deep fascia and the muscle layer of the abdominal wall, with the separation not exceeding the midline of the trunk.

**FIGURE 1:**
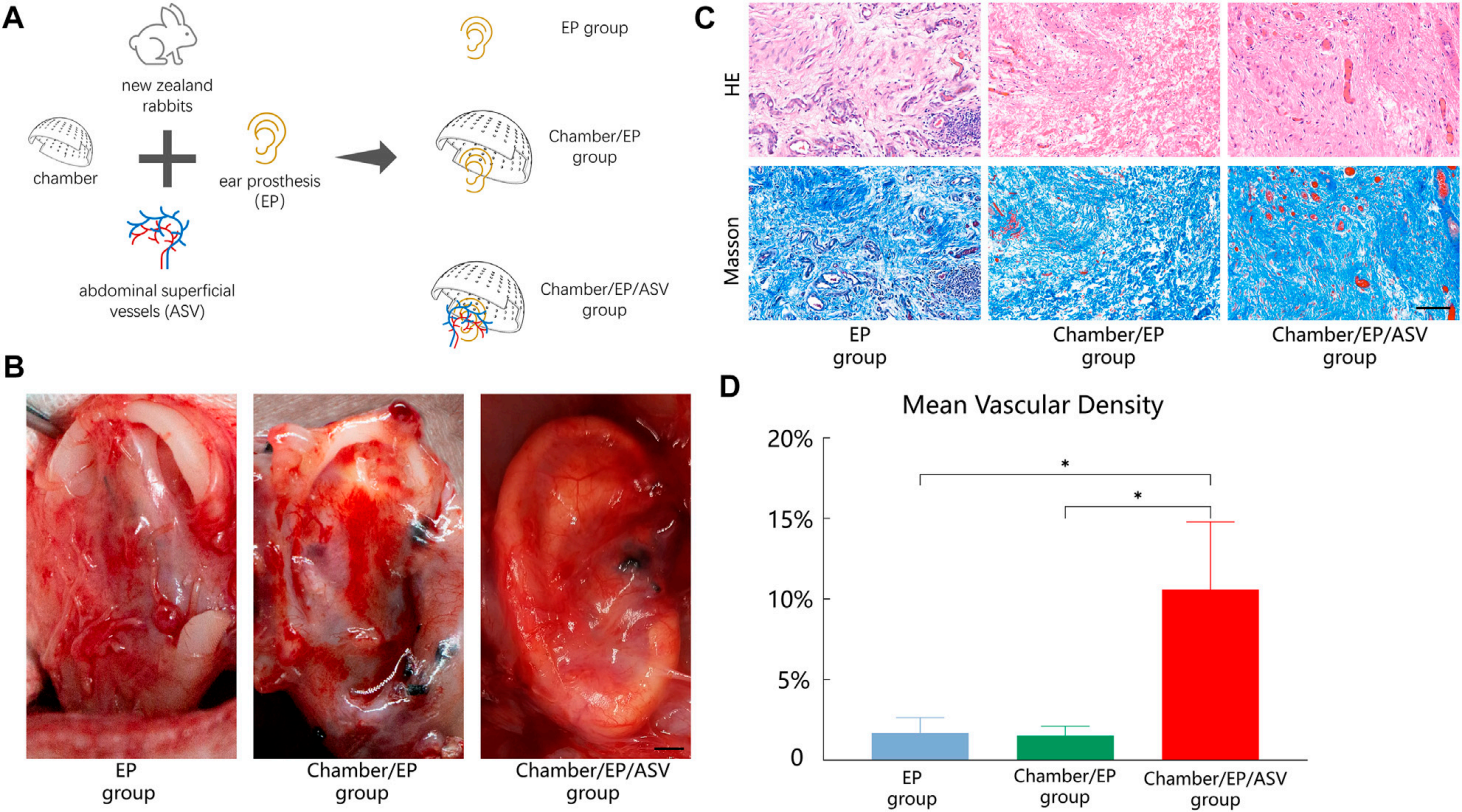
**(A)** Experimental grouping: 3 groups according to “whether to introduce ligated abdominal superficial vessels” and “whether to place Chambers”. EP group: only ear prosthesis; Chamber/EP group: pre-placed ear prosthesis and Chambers; Chamber/EP/ASV group: pre-placed ear prosthesis and Chambers, and introduced ligated abdominal superficial vessels. **(B)** A general view of the neonatal-EP compound, scale bar = 5 mm. 4 weeks postoperatively. In the EP group, the new fibrous tissue is seen to be incompletely wrapped around the ear prosthesis, with partial defects, irregular morphology, and pale tissue color. In the Chamber/EP group, thicker fibrous tissue was seen to roughly encapsulate the ear prosthesis, and the new fibrous tissue was paler at the elevated areas of the ear scaffold, such as the ear wheel, and the outline of the ear prosthesis was poorly displayed. In the Chamber/EP/ASV group, bright red, thin, vascularized neonatal tissue is seen to completely encapsulate the ear prosthesis, similar to the appearance of normal fascial tissue, and the shape of each part of the ear prosthesis is well visualized. A vascular network with an introduced cord-like ligated vascular bundle as the axis was seen. **(C)** H&E staining, Masson trichrome staining of neonatal fibrous tissue, scale bar = 100 um. At 4 weeks postoperatively, different degrees of lumen-like structures were seen in each group, and erythrocytes were visible in the lumen, suggesting mature vessels. Different degrees of inflammatory cell infiltration were also seen. Different degrees of inflammatory cell infiltration were also seen. H&E staining showed that most of the neoplastic vessels in the EP group and Chamber/EP group were distributed at the margins of the tissue and were accompanied by different degrees of inflammatory cell infiltration, showing chronic inflammation, especially in the EP group. In the Chamber/EP/ASV group, a large number of neovascularization with varying diameters was observed, and the lumen was filled with erythrocytes. Masson trichrome staining showed that the collagen fibers of EP group and Chamber/EP group were coarse, short and curly with disorganized arrangement, while the collagen fibers of Chamber/EP/ASV group were relatively neatly arranged. **(D)** Vascular density of neoplastic tissues. Using ImageJ analysis, the MVD was higher in the Chamber/EP/ASV group than in the EP group and Chamber/EP group (*p* < 0.05), and there was no difference in MVD between the EP group and the Chamber/EP group.

Grouping: All rabbits were randomly divided into three groups; 8 rabbits in each group (Figure 1A).

Ear Prosthesis placement Group (EP group): ear prosthesis placement. The ear prosthesis alone was placed into the separated cavity and fixed to the muscle layer of the abdominal wall with a 3–0 ligament.

Tissue Engineering Chamber and Ear Prosthesis Placement Group (Chamber/EP group): tissue engineering chamber and ear prosthesis placement. After fixing the ear prosthesis according to the method of the EP group, the perforated tissue engineering chamber was fixed to the muscle layer of the abdominal wall with 3–0 ligament and covered with the outer side of the ear prosthesis.

Tissue Engineering Chamber and Ear Prosthesis Placement with Abdominal Superficial Vessels Bundle Group (Chamber/EP/ASV group): The tissue engineering chamber and the ear prosthesis were placed with the abdominal superficial vessels bundle introduced. The abdominal superficial vessels are separated, the bundle is completely disconnected from the groin towards the abdomen, and the distal end is ligated. The bundle is 4.0 cm long, and the tip is 0.5 cm wide (care is taken not to damage the vessels to avoid vasospasm). After securing the ear prosthesis, the vascular bundle is transferred to the ear prosthesis and secured. The bundle should be free of traction and distortion. The tissue engineering chamber is then secured, and the bundle is passed through the hole in the margin of the chamber. Ensure that the ear prosthesis and the chamber are securely fixed to avoid movement of the device after the rabbit awakes, which could cause the vascular bundle to be stuck and thus embolized.

Postoperative management: The skin incision was closed with simple interrupted sutures with 6–0 ligament, leaving no dead space. To prevent infection, penicillin was given 30 min before surgery and 3 days after surgery. Each rabbit was operated on bilaterally. Dead and infected rabbits at the surgical site were excluded.

### Neonatal tissue acquisition and observation

Four weeks after surgery, the rabbits were anesthetized and entered through the original surgical incision to observe the placements and the newborn tissues.

### Implantation test (vascularized fascial function verification test)

After 4 weeks of surgery, the animals of Chamber/EP/ASV group (8 rabbits) were anesthetized, accessed through the existing surgical incision, and the tissue of the surgical cavity was gently separated to expose the chambers. The chambers were separated and removed, and a full-thickness skin slice of approximately 4 cm × 4 cm was taken next to the incision and used for the *in situ* skin graft test. The skin incision was closed with 6–0 silk wire, and the skin defect at the skin extraction site was treated with *in situ* skin grafting: 6–0 silk wire was used to fix the skin slice, and 6–0 silk wire was used for local pressure bandaging, and the graft was opened after 1 week to observe the survival of the skin slice.

### Histomorphological observation

The appearance of the neonatal tissue-ear prosthesis compound, the level of vascularization, and the patency of the abdominal superficial vessels were observed. H&E staining and Masson trichrome staining of tissue sections: The neonatal tissue-ear prosthesis compound was removed intact, fixed in 4% paraformaldehyde, paraffin-embedded and sectioned, and subjected to H&E staining, Masson trichrome staining, and immunohistochemical staining (Ki67, CD31, type I collagen, type II collagen). For each sample, one section was made in each of the tissue block’s upper, lower, left, right, and central regions. The sections were subjected to H&E staining for vascular density counting (luminal structures containing erythrocytes were determined as mature vessels). Three fields of view were randomly selected for each H&E staining section to assess vascular density (Microvascular Density, MVD). The average vessel density in three fields of view of each section was used as the vessel density of that section. The average vascular density of the above five sections was used as the vascular density of the sample (Scherer et al., 2008).

### Micro-CT scan and 3D reconstruction analysis

#### Microfil contrast-medium perfusion and specimen processing

The heparin sodium saline (500 U/100 ml) was perfused through the abdominal aorta and inferior vena cava until the inferior vena cava effluent was clarified. Microfil perfusion solution was configured according to the instructions. The perfusion solution was injected slowly and uniformly through the abdominal aorta (flow rate about 20 ml/min) until the inferior vena cava saw yellow perfusion flow. Then the inferior vena cava and abdominal aorta were ligated and stored at 4°C until the next day for collection. The neoplastic tissue-ear prosthesis compound was obtained, fixed in 4% paraformaldehyde for 2 days, washed three times with PBS, and stored in 75% alcohol.

#### Micro-CT scan and 3D reconstruction of blood vessels

The samples were subjected to a Micro-CT scan and vascular 3D reconstruction, and the accompanying software obtained the total volume of tissue and total volume of blood vessels. (Koshiwa Biological Corporation provided micro-CT scan and 3D reconstruction of blood vessels).

### Statistical analysis of data

Data were analyzed using R software (version 4.1.2), one-way ANOVA was used to compare groups, and differences were considered at *p* < 0.05.

## Results

### Histomorphology

The samples were taken 4 weeks after surgery. In all groups, new pericardial tissue could be seen on the surface of the inserted ear prosthesis. The new pericardial tissue morphology and degree of vascularization varied among the groups (Figure 1B).

In Chamber/EP group and Chamber/EP/ASV group with the tissue engineering chamber, an envelope was formed on the outer surface of the chamber, and the envelope tissue was attached to the tissue inside the chamber through the hole on the surface of the chamber. Removing the envelope and the chamber, clear yellowish fluid was seen to flow out from the chamber.

In the EP group, the new fibrous tissue could not completely wrap around the ear prosthesis, the envelope morphology was incomplete, and the new tissue was pale in color and tough in texture. In Chamber/EP group, the new fibrous tissue was able to wrap the ear prosthesis, and the new tissue was at the elevation of the ear prosthesis (e.g., the auricle, etc.), with a paler color and tough texture. In Chamber/EP/ASV group, the introduced cords of ligated vascular bundles were seen pulsating, and the new fibrous tissue was completely wrapped around the ear prosthesis. The new tissue was bright red in color, softer in texture, and contained abundant enveloping vessels, with the introduced abdominal superficial vessels as the axis, forming a vascular network that resembled the appearance of normal fascia flap tissue (Figure 1B, the image on the right side of Figure 1B shows the pattern of blood vessel growth around the ear prosthesis in the chamber of chamber/ep/asv group.). The tissue was taken 4 weeks after surgery. H&E staining showed different degrees of fibrous tissue neogenesis, angiogenesis, and inflammatory cell infiltration in all groups. Neovascularization in EP group and Chamber/EP group was mostly distributed around the new fibrous tissue, with neovascular structures resembling veins and different degrees of inflammatory cell infiltration. This was similar to the manifestation of chronic inflammation, especially in the EP group. In Chamber/EP/ASV group, enriched neovascularization and fibrous tissue could be observed. Vessels were heterogeneous in diameter, artery-like or vein-like structures were seen, and the lumen was filled with red blood cells, indicating mature vessels. Masson trichrome staining observation showed that the fibrous tissues in EP group and Chamber/EP group were less disorganized, with thick and short fibers and curled. In contrast, the neonatal fibers in Chamber/EP/ASV group were relatively neatly arranged (Figure 1C).

Analyzing the microvascular density (MVD) of neonatal tissues in each group using ImageJ software, the MVD of the EP group, Chamber/EP group, and Chamber/EP/ASV group were approximately 1.74% ± 1.48%, 1.69% ± 0.64%, and 12.13% ± 2.83%, respectively. The MVD of the Chamber/EP/ASV group was significantly higher than that of the other two groups, but no significant difference in MVD was seen between the EP group and the Chamber/EP group (*p* < 0.05, Figure 1D).

**FIGURE 2:**
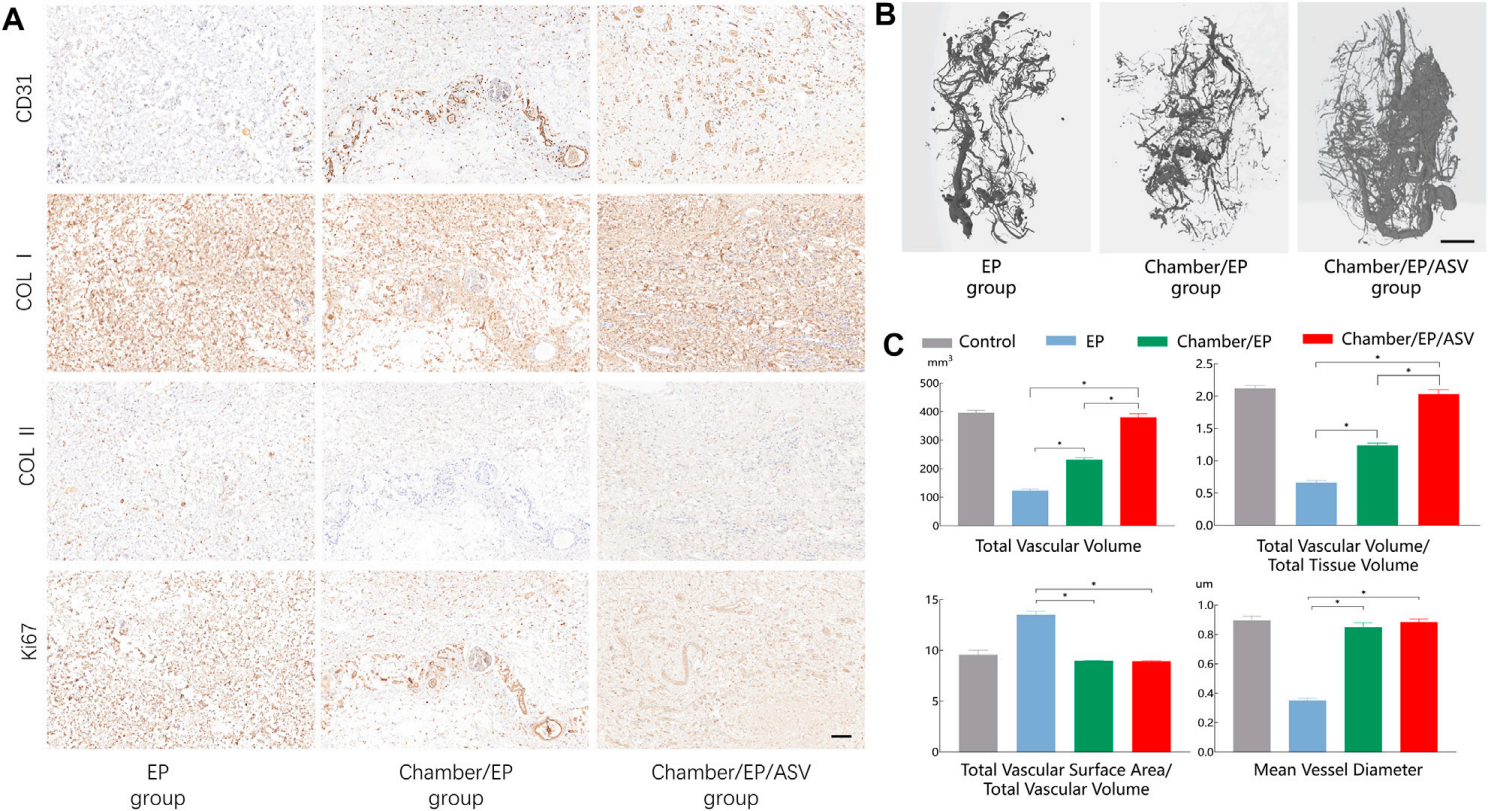
**(A)** Immunohistochemical staining, scale bar = 100um. CD31 is labeled with endothelial cells, indicating the degree of vascularization, revealing that the Chamber/EP/ASV group is well vascularized. Type I collagen, type II collagen staining shows the type of collagen in the neoplastic tissue. ki67 shows the active proliferation of the tissue, and the Chamber/EP/ASV group is actively proliferating. **(B)** Micro-CT scan 3D reconstruction, scale bar = 5 mm. In the 3D reconstruction after Micro-CT scan, the vascular network was visible on the outer surface of the new fibrous tissue in all groups, and small branches of blood vessels were also seen extending to the inner part of the tissue. Compared with the Chamber/EP/ASV group, the blood vessels in the EP group and Chamber/EP group were smaller and sparser. In the Chamber/EP/ASV group, trailing budding vessels emanating from the placed ligated vascular bundles were also seen, which were numerous and dense with varying thickness. **(C)** Vascular analysis after 3D reconstruction of Micro-CT scan. Total vessel volume (TVV), total vessel volume/total tissue volume (TVV/TV) indicated that the Chamber/EP/ASV group was more vascularized than the other two groups (*p* < 0.05), and there was no difference compared with the control group. Total vascular surface area/total vascular volume (TVS/TVV), mean vascular diameter indicated that the mean vascular diameter was greater in Chamber/EP group and Chamber/EP/ASV group than EP group (*p* < 0.05) and did not differ from the control group. However, there was also no difference between Chamber/EP group and Chamber/EP/ASV group.

Further immunohistochemical staining was performed to understand the vascularization and fibrosis of the neoplastic tissue. Labeling of endothelial cells (CD31) showed tubular-like neovascularization, with a high degree of vascularization in the neonatal tissue in the Chamber/EP/ASV group, consistent with the H&E staining results. Type I collagen and type II collagen staining was used to determine the type of fibrin within the neonatal tissues. Histochemical staining suggested that the main collagen fibril type in the neoplastic tissue was type I collagen, consistent with the reported collagen types in normal human fibrous tissue (Figure 2A).

### Micro-CT scan 3D reconstruction after vascular analysis

Microfil perfusion, sampling, and Micro-CT angiography reconstruction were performed 4 weeks after the experiment. The vascular network was visible on the surface of the new tissues in all groups and extended to the interior of the tissues through small branches. Still, the vascular network on the surface of the tissues in the EP group and Chamber/EP group was smaller and sparser compared with Chamber/EP/ASV group. Meanwhile, sprawling, budding vessels, which were numerous and dense with varying thickness, were visible inside the Chamber/EP/ASV group emanating from the tips of the placed vessels. This angiogenesis pattern appeared similar to the pattern of vascular budding reported in adult individuals. (Figure 2B shows the vascularization of the 3D micro-CT reconstruction images for the different subgroups. Note that the interruption in imaging of the distal end of the vessel is due to a break in the perfusion cast).

By analyzing the relevant factors of the reconstructed vascular images, the results showed that Chamber/EP/ASV group was higher than the other groups in terms of total vessel volume and total vessel volume/total tissue volume (*p* < 0.05), indicating that the degree of vascularization in Chamber/EP/ASV group may be higher than the other two groups. And there was no significant difference in the degree of vascularization in the Chamber/EP/ASV group compared with normal fascial tissue. The total surface area through vessels/total vessel volume and mean diameter of vessels in the Chamber/EP group and Chamber/EP/ASV group were significantly greater than those in the EP group (*p* < 0.05); however, no significant difference between Chamber/EP group and Chamber/EP/ASV group was seen. The mean vessel diameters in Chamber/EP group and Chamber/EP/ASV group were likely higher than those in the EP group. Although the mean vessel diameters of the Chamber/EP group and Chamber/EP/ASV group were similar, considering that the degree of vascularization in the Chamber/EP/ASV group was higher than that in Chamber/EP group, the overall degree of vascular supply in the Chamber/EP/ASV group could be assumed to be better than that in Chamber/EP group (Figure 2C).

**FIGURE 3:**
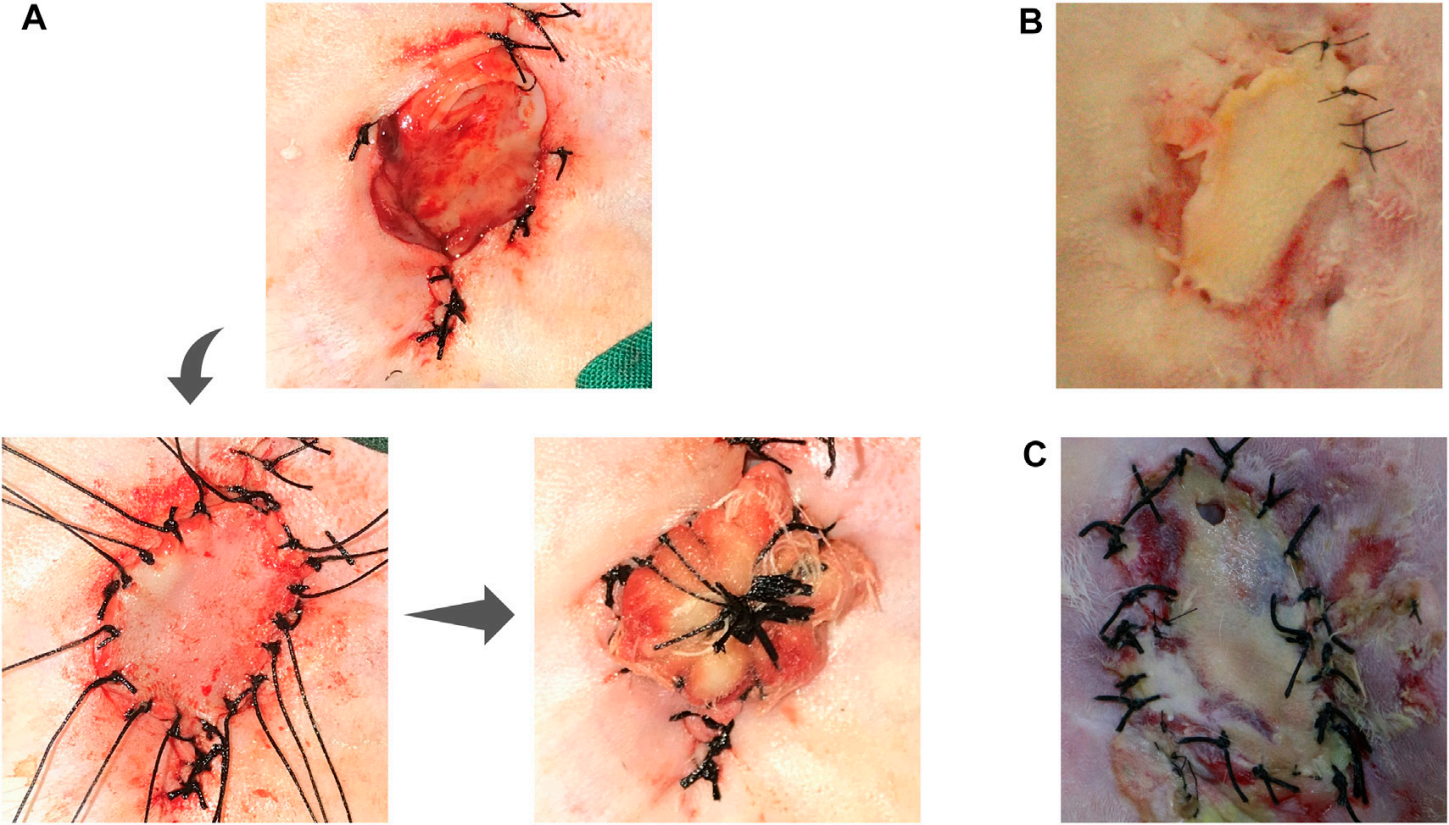
*In-situ* implant test. **(A)** shows the steps of *in situ* full-thickness skin graft: Primary incision approach, separating the chamber, exposing the ear prosthesis and its surface fascia, taking full-thickness skin slice for *in situ* skin grafting; *in situ* skin grafting, fixing the skin slice; local skin slice with pressure bandage; **(B)**: unpacking the bandage auxiliary material 1 week after grafting, showing the survival of the grafted skin slice; **(C)**: Skin slice necrosis appearance: the skin slice is pale and presents a contracted state, lack of blood supply, and local perforation.

The value of vascularized fascial tissue in promoting graft survival was further supported by *in situ* grafting tests. The survival of the graft was observed at 1 week postoperatively through the regular full-thickness skin grafting procedure, which initially showed the possibility of constructing vascularized fascial tissue for graft repair (Figures 3A,B). In our procedure, we set up the procedure to open the packing 1 week after implantation to check the survival of the graft, so what we could observe was almost always the end condition after implantation, and there was absolutely no opportunity to see the development of the graft after implantation. Thus, in our experiment, the outcome of the graft was either survival or failure, with failure often being a combination of skin infection, necrosis, and local skin perforation (Figure 3C), and the survival rate of the graft was 62.5% (5/8). Note that in Figure 3, we show the skin grafting of the Chamber/EP/ASV group animals, while in the reality, we also performed *in situ* full-thickness skin grafting in the other two groups, but at the beginning of the grafting test, there were three consecutive graft failures, which we aborted for animal welfare concern in the other two groups.

## Discussion

Our study showed that a large amount of well-vascularized fascial tissue could be obtained by introducing blood vessels into the tissue-engineered chamber of a preplaced ear prosthesis. With “Introduction of blood vessels into the tissue-engineered compartment of the preplaced ear prosthesis,” the amount of fascial tissue obtained was not only large but also well vascularized compared to “Placement of ear prosthesis alone” and “Placement of only a composite structure of ear prosthesis and tissue engineering chamber without the introduction of blood vessels.” The above method provides an idea for the source of soft tissue coverings for ear reconstruction surgery.

Ear reconstruction, especially auricular reconstruction, has always been one of plastic surgery’s most challenging aspects of body-surface organ reconstruction. A moderately elastic ear scaffold and soft tissue to cover the surface of the ear scaffold should be available for ear reconstruction. The soft tissue covering the ear scaffold should be as similar in color and texture as possible to the surrounding skin and have an adequate blood supply to avoid skin flap necrosis, which could result in the exposure of the ear scaffold. For patients with external ear defects caused by special circumstances such as burns, trauma, or secondary ear reconstruction, or for patients suffering from localized peri-auricular conditions that limit ear reconstruction, the absence of available soft tissue makes ear reconstruction work more difficult (Bhandari, 1998). As for the reconstruction methods for auricular deformities or defects caused by trauma or burns, no systematic and mature treatment protocols have been established in clinical practice. The choice of surgical option and the ease of surgery depend on the availability and quality of the soft tissue covered by the ear stent. Common methods of obtaining soft tissue for the ear scaffold include a contralateral free superficial temporal fascial flap, an upper arm medial flap, or an expanded flap obtained with a dilator. However, these methods are associated with poor blood supply to the reconstructed ear, susceptibility to postoperative necrosis, and bloated tissue with poor anatomical morphology. In this experiment, a graftable fasciocutaneous ear prosthesis complex was preplaced to provide a reliable blood supply to the soft tissue covered with an ear scaffold for further surgery and a practical solution for patients with auricular defects caused by trauma or burns (Selcuk et al., 2014; Bos et al., 2016).

Pericapsular tissue flaps have been used in clinical reconstructive surgery since the 1990s (Bengtson et al., 1993; Heymans et al., 1993). However, the lack of vascular supply limits the clinical application of pericapsular tissue flaps. Attempts have been made to obtain a pericapsular tissue flap by implanting a constructed tissue-engineered product through a tissue engineering approach. However, this method is inadequate for the long-term survival of the pericapsular flap tissue due to the long time required and the early infiltration of the surrounding tissue alone (Tissue transfer after implantation of an envelope capsule tissue flap, or tissue engineering product constructs, both lead to graft failure due to lack of axial cardiovascular supply) (Eto et al., 2012; Kato et al., 2014; Dew et al., 2015; Mashiko and Yoshimura, 2015). By constructing tissue-engineered chambers *in vivo* with the addition of vascular growth factors, although more fibrous tissue can be obtained, it may cause risks such as uncontrolled vascular growth. Kataras et al. constructed vascularized cartilage-skin flap complexes by placing autologous cartilage or artificial material in rabbits and performed transplantation experiments after 2 weeks of experimentation, found that the survival rate of vascularized cartilage-skin flap complexes was roughly similar to that of conventional axial flaps (Karatas et al., 2000). *In vitro* tissue engineering chambers with preplaced vessels have been widely used and have led to *in vivo* construction of “vascularized tissue engineering chambers" (Karatas et al., 2000; Beier et al., 2009). In this experiment, we introduced a new concept of vascularized tissue engineering chamber based on the previous work, and pre-constructed vascularized fascial flap ear prosthesis complex by placing 3D printed ear prostheses with preplaced vascular tips inside the tissue engineering chamber and obtained richly vascularized fascial flaps with tips.

As for the mechanism of formation of fibrous tissue and vascularity, we believe there are several aspects. First, separating the superficial abdominal wall vessels and the surgical gap causes unavoidable tissue damage, even when sufficient care is taken. This damage may cause corresponding changes in relevant cytokines, initiating the process of angiogenesis and tissue repair. Secondly, the formation of gradient hypoxia inside the tissue engineering chamber may promote the upregulation of hypoxia-inducible factor expression and the expression of a series of regulatory factors, such as VEGF, which activates endothelial cells and initiates the angiogenic process (Thomas et al., 2008; Peng et al., 2014). Finally, when the ear prosthesis and the small chamber are placed into the organism, a foreign body reaction is induced, leading to the release of inflammatory factors and fibroblast chemotaxis and differentiation into myofibroblasts, which form the main cellular components of the new connective tissue, while the hypoxic microenvironment of the surgical cavity induces the vascular growth of the surrounding tissue into the vascularized new connective tissue.

There are several lessons to be learned from this experiment. First, tissue engineering chambers constructed of silicone materials have a greater advantage over polycarbonate materials in maintaining vascular patency, angiogenesis, and tissue formation. Meanwhile, tissue-engineered chambers with perforated surfaces can better promote the growth of tissues within the chamber (Cronin et al., 2004). Secondly, the construction of rigid non-collapsible chambers can induce cell migration, proliferation, and differentiation by causing local stress changes (Rosenfeld et al., 2016; Dorland and Huveneers, 2017). Again, the distal ligation of arteriovenous bundles may provide a more effective use of vascularization. Another interesting phenomenon was that Micro-CT scans revealed the presence of tree-root-like sprouting vessels in all groups, especially in the Chamber/EP/ASV group, which is consistent with the literature reporting that the main form of angiogenesis in adult individuals is the sprouting type and suggests that the introduced vascular tips not only survive in the new tissue but also become its main source of blood supply. The above phenomenon may be due to the ligation of the distal end of the abdominal superficial vessels, which leads to the formation of turbulence and increased Circumferential Walls Stress and Fluid Shear Stress after the blood reaches the ligated end. The “turbulent flow” and “increased fluid shear stress” in the blood vessel are the main initiating factors for activation of vascular endothelial cells, which can promote vascular sprouting and collateral angiogenesis. Therefore, from a clinical and practical point of view, distal arteriovenous bundle ligation is an effective method of vascularization (Galie et al., 2014; Zhang et al., 2022; Zhao et al., 2023).

For the clinical vision of the application of this method, we believe that there are several aspects. First, our approach to soft tissue fascia construction resolves clinical cases where subcutaneous tissue is lacking, especially (but not limited to) soft tissue defects caused by trauma, burns, etc. Secondly, the method of constructing a “scaffold-soft tissue complex” in the distal septum allows for a staged reconstructive surgery that is not based on the soft tissues surrounding the reconstructive site, allowing for a flexible surgical design in the first stage of surgery. Finally, the successful construction of the “ear prosthesis-fascia complex” is a precursor to the final construction of the “ear prosthesis-fascia-skin complex”. The study of the “ear prosthesis-fascia-skin complex” may make it possible to turn a complex multi-stage ear reconstruction surgery after trauma or burns into a simple two-stage surgery (the “ear prosthesis-fascia-skin complex” construction surgery). The “ear prosthesis-fascia-skin complex” grafting procedure will help the above-mentioned patients and provide a reference for similar organ reconstructive surgery.

Our study also has some limitations, which are the main directions of our next phase of research. First, we will continue to conduct grafting tests to further validate the role of vascularized Fascia-Prosthesis Compound in repair by increasing the number of samples. Secondly, we will try to focus not only on the survival of the skin slice but also on the morphology of the auricle at the implant site in the grafting test to better fit the clinical application scenario; finally, we will test the main vascular growth factors related to the different stages of the vascularized Fascia-Prosthesis Compound construction to determine the major factor types. By identifying the main types of factors that play a role, it may be possible to further regulate the degree of vascularization and improve the survival rate of the graft test.

## Conclusion

A well-vascularized fascia-ear prosthesis complex can be obtained by introducing ligated vascular bundles through a preplaced tissue engineering chamber at the ear prosthesis. It provides a new idea for the source of soft tissue fascia in clinical ear reconstruction surgery.

## Data Availability

The raw data supporting the conclusion of this article will be made available by the authors, without undue reservation.
